# Supplementary material for: Comparative Study of the Myocardium of Patients from Four COVID-19 Waves
Source: Diagnostics (Basel). 2023 May 7;13(9):1645. doi: 10.3390/diagnostics13091645 (PMC10178873; doi:10.3390/diagnostics13091645)
Supplement: Supplementary file 1 [file diagnostics-13-01645-s001.zip › Appendix S2.pdf]

Appendix S2

Clinical and laboratory parameters of deceased patients with immunohistochemical diagnosis of viral myocarditis

№	Gender , age	Anamnestic data and symptoms till the moment of hospitalization	Laboratory data	Simpson left ventricular ejection fraction	Lung lesions on computed tomography till the moment of hospitalization (in %)	Concomitant pathology	Arrhythmias and conduction disorders
1	F, 62	Dry cough<br>Chest congestion<br>Dyspnea<br>Anosmia<br>Time from onset of symptoms and until staying in hospital was 4 days	CRP 50 (mg/l)<br>Lymphocytes 0•56 x10^9/l<br>D-dimer 4900 (ng/ml)<br>Procalcitonin 0•072 (ng/ml)<br>Troponin 0•39 (ng/ml)	Not carried out	90%	Type 2 diabetes<br>Hypertensive heart disease<br>Chronic obstructive pulmonary disease	Left Anterior Fascicular Block
2	M, 71	Dry cough<br>Diarrhea<br>Tiredness<br>Time from onset of symptoms and until staying in hospital was 4 days	Ferritin 3175 (mcg/l)<br>CRP 155 (mg/l)<br>Lymphocytes 0•6 x10^9/l<br>D-dimer 1475 (ng/ml)<br>Procalcitonin 0•45 (ng/ml)<br>Troponin 15•0 (ng/ml)	47%	85%	Type 2 diabetes<br>Hypertensive heart disease<br>Myocardial ischemia	Atrial extrasystole
3	M, 61	Chest congestion<br>Dyspnea<br>No information about symptoms	Ferritin 2000 (mcg/l)<br>CRP 250 (mg/l)<br>Lymphocytes 0•7 x10^9/l<br>D-dimer 1160 (ng/ml)<br>Procalcitonin 0•94 (ng/ml)<br>Troponin 0•74 (ng/ml)	Not carried out	80%	Type 2 diabetes<br>Hypertensive heart disease<br>Myocardial ischemia	Atrial fibrillation<br>Ventricular extrasystoles
4	F, 82	No information due to extremely serious condition	Ferritin 378 (mcg/l)<br>CRP 94 (mg/l)<br>Lymphocytes 0•8 x10^9/l<br>D-dimer 700 (ng/ml)<br>Procalcitonin 3•96 (ng/ml)<br>Troponin 2•97 (ng/ml)	30%	10%	Hypertensive heart disease<br>Cerebrovascular disease	Absent
5	M, 59	Dry cough<br>Chest congestion<br>Dyspnea<br>Sweating<br>Time from onset of symptoms and until staying in hospital was 3 days	Ferritin 420 (mcg/l)<br>CRP 390 (mg/l)<br>Lymphocytes 0•03 x10^9/l<br>D-dimer 1550 (ng/ml)<br>Procalcitonin 0•05 (ng/ml)<br>Troponin 0•02 (ng/ml)	Not carried out	85%	Hypertensive heart disease<br>Pulmonary sarcoidosis	Right Bundle Branch Block
6	M, 62	Tiredness<br>Dry cough<br>No information about symptoms	Ferritin 2000 (mcg/l)<br>CRP 81•4 (mg/l)<br>Lymphocytes 1•58 x10^9/l<br>D-dimer 1470 (ng/ml)<br>Procalcitonin 0•42 (ng/ml)<br>Troponin 1•21 (ng/ml)	Not carried out	70%	Hypertensive heart disease<br>Myocardial ischemia<br>Cerebrovascular disease<br>Obesity	Right Bundle Branch Block
7	F, 68	No information due to extremely serious condition	CRP 161 (mg/l)<br>Lymphocytes 0•57 x10^9/l	Not carried out	64%	Hypertensive heart disease	Right Bundle Branch Block<br>Atrial fibrillation

			D-dimer 1300 (ng/ml)<br>Procalcitonin 0•35 (ng/ml)<br>Troponin 0•12 (ng/ml)			Myocardial ischemia	
8	F, 59	Tiredness<br>Dry cough<br>Dyspnea<br>Headache<br>Time from onset of symptoms and until staying in hospital was 12 days	Ferritin 492 (mcg/l)<br>CRP 41 (mg/l)<br>Lymphocytes 0•43 x10^9/l<br>D-dimer 3060 (ng/ml)<br>Procalcitonin 4•51 (ng/ml)<br>Troponin 0•1 (ng/ml)	70%	90%	Type 2 diabetes<br>Hypertensive heart disease	Right Bundle Branch Block<br>Atrial fibrillation
9	V, 58	Wet cough<br>Dyspnea<br>Tiredness<br>Chest congestion<br>Time from onset of symptoms and until staying in hospital was 7 days	Ferritin 1124 (mcg/l)<br>CRP 113•12 (mg/l)<br>Lymphocytes 0•48 x10^9/l<br>D-dimer 1000 (ng/ml)<br>Procalcitonin 0 (ng/ml)<br>Troponin 0•1 (ng/ml)	65	75	Absent	Absent
10	V, 73	Wet cough<br>Tiredness<br>Diarrhea<br>Dyspnea<br>Sore throat, nasal congestion<br>Time from onset of symptoms and until staying in hospital was 5 days	Ferritin 1233 (mcg/l)<br>CRP 170•63 (mg/l)<br>Lymphocytes 1•32 x10^9/l<br>D-dimer 440 (ng/ml)<br>Procalcitonin 0•623 (ng/ml)<br>Troponin 0•1 (ng/ml)	Not carried out	56	Hypertensive heart disease<br>Myocardial ischemia<br>Rheumatoid arthritis	Absent
11	V, 84	Wet cough<br>Tiredness<br>Headache<br>Dyspnea<br>Time from onset of symptoms and until staying in hospital was 7 days	Ferritin 106 (mcg/l)<br>CRP 50•49 (mg/l)<br>Lymphocytes 0•61 x10^9/l<br>D-dimer 350 (ng/ml)<br>Procalcitonin 0•148 (ng/ml)<br>Troponin 0•1 (ng/ml)	48	65	Hypertensive heart disease<br>Myocardial ischemia<br>Type 2 diabetes	Atrial fibrillation
12	V, 86	Wet cough<br>Tiredness<br>Headache<br>Dyspnea<br>Sweating<br>Time from onset of symptoms and until staying in hospital was 8 days	Ferritin 419•5 (mcg/l)<br>CRP 34•69 (mg/l)<br>Lymphocytes 0•83 x10^9/l<br>D-dimer 210 (ng/ml)<br>Procalcitonin 0•083 (ng/ml)<br>Troponin 0•157 (ng/ml)	55	20	Hypertensive heart disease<br>Myocardial ischemia<br>Type 2 diabetes<br>Cerebrovascular disease	Atrial fibrillation<br>Ventricular extrasystoles<br>Left Anterior Fascicular Block
13	V, 82	Wet cough<br>Tiredness<br>Nasal congestion<br>Dyspnea<br>Time from onset of symptoms and until staying in hospital was 11 days	Ferritin 263•3 (mcg/l)<br>CRP 25•7 (mg/l)<br>Lymphocytes 0•25 x10^9/l<br>D-dimer 190 (ng/ml)<br>Procalcitonin 0•033 (ng/ml)<br>Troponin 0•668 (ng/ml)	70	60	Hypertensive heart disease<br>Myocardial ischemia<br>Type 2 diabetes	Absent
14	V, 59	Dyspnea<br>Tiredness<br>Edema of the legs and face<br>Time from onset of symptoms and until staying in hospital was 12 days	CRP 27•8 (mg/l)<br>Lymphocytes 0•72 x10^9/l<br>D-dimer 15092 (ng/ml)<br>Procalcitonin 4•77 (ng/ml)<br>Troponin 0•059 (ng/ml)	55	Not carried out		Atrial fibrillation
